# Web-Based Patient Segmentation in Finnish Primary Care: Protocol for Clinical Validation of the Navigator Service in Patients With Diabetes

**DOI:** 10.2196/20570

**Published:** 2020-11-02

**Authors:** Riikka Riihimies, Elise Kosunen, Tuomas Koskela

**Affiliations:** 1 Faculty of Medicine and Health Technology Tampere University Tampere Finland; 2 Health Center Valkeakoski Finland; 3 Center of General Practice Pirkanmaa Hospital District Tampere Finland

**Keywords:** patient segmentation, equality, health care services, coordination of care, primary care, Navigator, psychometric properties, questionnaires, eHealth

## Abstract

**Background:**

An aging population and increasing multimorbidity challenge health care systems worldwide. Patient segmentation aims to recognize groups of patients with similar needs, offer targeted services to these groups, and reduce the burden of health care. In this study, the unique Finnish innovation Navigator, a web-based service for patient segmentation, is presented. Both patients and health care professionals complete the electronic questionnaire concerning patients’ coping in everyday life and health state. Thus, it considers the patient perspective on self-care. One of four customership-strategy (CS) groups (self-acting, community, cooperating, and network) is then proposed in response to the answers given. This resulting strategy helps both professionals to coordinate patient health care and patients to utilize appropriate health services.

**Objective:**

This study aims to determine the feasibility, validity, and reliability of the Navigator service in the segmentation of patients with diabetes into four CS groups in a primary care setting. Patient characteristics concerning demographic status, chronic conditions, disabilities, health-related quality of life, and well-being in different CS groups will be described. We hypothesize that patients in the network group will be older, have more illnesses, chronic conditions or disabilities, and require more health care services than patients in the self-acting group.

**Methods:**

In this mixed methods study, data collection was based on questionnaires (user experience of Navigator, demographic and health status, World Health Organization Disability Assessment Schedule 2.0, EuroQol 5D, Wellbeing Questionnaire 12, and the Diabetes Treatment Satisfaction Questionnaire) issued to 300 patients with diabetes and on user-experience questionnaires for and semistructured focus-group interviews with 12 nurses. Navigator-database reports and diabetes-care values (blood pressure, BMI, HbA1c, low-density lipoprotein, albumin-creatinine, smoking status) were collected. Qualitative and descriptive analyses were used to study the feasibility, content, concurrent, and face validity of Navigator. While criterion and concurrent validity were examined with correlations, reliability was examined by calculating Cohen kappa and Cronbach alpha. Construct validity is studied by performing exploratory-factor analysis on Navigator data reports and by hypothesis testing. The values, demographics, and health status of patients in different groups were described, and differences between groups were studied by comparing means. Linear regression analysis was performed to assess which variables affect CS group variation.

**Results:**

Data collection was completed in September 2019, and the first feasibility results are expected by the end of 2020. Further results and publications are expected in 2021 and 2022.

**Conclusions:**

This is the first scientific study concerning Navigator’s psychometric properties. The study will examine the segregation of patients with diabetes into four CS groups in a primary care setting and the differences between patients in groups. This study will assist in Navigator’s further development as a patient segmentation method considering patients’ perspectives on self-care. This study will not prove the effectiveness or efficacy of Navigator; therefore, it is essential to study these outcomes of separate care pathways.

**International Registered Report Identifier (IRRID):**

DERR1-10.2196/20570

## Introduction

Primary health care systems should deliver services equally for every patient, but an aging population and increasing multimorbidity challenge the capacity of health care systems worldwide [1-5]. Treatment of multiple medical conditions in different health care organizations may lead to fragmentation and incomplete coordination of care, and inefficiency, ineffectiveness, and inequality as unintended consequences of fragmented care [6,7]. Patients’ unmet needs lead to poor health outcomes, inappropriate services, and rising costs [1,8,9]. Inequalities in health care appear owing to socioeconomic differences in health and the use of health care services [10-14]. In the 1970s, this was described as The Inverse Care Law [15-17]. One size does not fit all in health services, and one care pathway is not suitable for all patients.

Patient segmentation is an approach that aims to recognize groups of patients with similar health care needs and help care providers to develop services targeted to these groups [18-20]. The origin of patient segmentation was provided in 1970 by Dr Garfield at Kaiser Permanente when recognizing groups of the well, the worried well, early sick, and sick patients were introduced in the new approach to medical care delivery [21]. Another aspect of patient segmentation derives from the field of business and marketing, where, in addition to medical conditions, patients’ needs, willingness, and self-efficacy to care are presented to be combined with the production logic of health care services [22,23].

Methods for patient segmentation are either data-driven, where data from different databases and/or electronic health records (EHR) are collected and statistically analyzed [24,25], or expert-driven, where an expert defines the criteria for segmentation [25,26]. The Senior Segmentation Algorithm is a segmentation tool developed at Kaiser Permanente and implemented in the EHR, utilizing EHR data, risk scores, and indicators [27]. Simple Segmentation Tool (SST) is for segmenting the aging population in Singapore and administered by the clinician [28,29]. Different patient segments have been based on medical condition or clinical criteria [30], patient utilization of services [31], and costs of health care [9,32], risk algorithms [33], difficulties in functioning [34], or a combination of factors [29,35]. However, none of these segmentation methods consider personal needs, values, or patients’ conditions in individual care. When patient-centered care and health services are planned, patient’s self-efficacy and everyday coping should be objectives [36-38]. Moreover, developing separate care pathways for different patient segments could help allocate health care professional resources to the most vulnerable patients and develop and target electronic services to patients capable of managing and navigating in health services.

**Figure 1:**
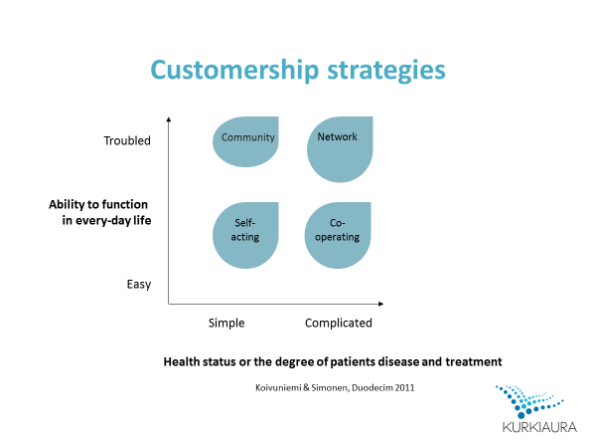
Dimensions of Navigator and four CS groups.

In Finland, an innovation for patient segmentation has been developed that, remarkably, considers the patients’ perspectives regarding their everyday life and self-care resources. Navigator (*Suuntima* in Finnish) is a web-based service for use at appointments. Both patients and health care professionals complete their electronic questionnaires during the conversation. While patients’ questions measure their ability to function in everyday life, questions for professionals measure patients’ health status or the degree of their diseases and treatments (Figure 1). As a result of these questions, one of four different customership-strategy (CS) groups is proposed: self-acting, community, cooperation, or network. Care pathways differ for each group, thus guiding professionals in coordinating patients’ health services as appropriately as possible. The CS group-related care pathway aims to empower patients in self-care by helping them to utilize appropriate health services. The CS group does not guide a patient’s medical treatment. The basis of “customership strategies” is in business and marketing; thus, the same terminology has been used here. In health care, the patient is the customer and the nurse or physician the professional.

During Navigator’s development, the model was piloted among patients with acute myocardial infarct and patients suffering from alcohol or drug abuse or mental disorders. Segregation of these patients left 60%-66% in self-acting, 10%-20% in the community, 10%-25% in cooperating, and 9%-10% in the network group.

Though Navigator is a general service and suitable to be used with patients with any chronic condition, diabetes was chosen as an example disease because it is a major, seriously complicated, and expensive chronic condition both in Finland and worldwide, requiring multiple medical services [39,40]. In 2019, diabetes was diagnosed in 463 million patients between 20-79 years, and it is estimated that the prevalence increases 51% globally, meaning 700 million patients with diabetes in 2045 [39]. Multifactorial barriers in patients’ diabetes management are related to adherence and knowledge about diabetes, cultural aspects, comorbidities, financial resources, and social support [41]. Moreover, barriers in provider and health care systems influence self-care, and self-care barriers are related to complications with type 2 diabetes [42]. Thus, segmenting the vast population of patients with diabetes is essential, and Navigator service might help to recognize and exceed barriers in diabetes management.

This study is the first to address the Navigator service’s psychometric properties and the segmentation of patients into CS groups. This study aims to examine the feasibility of Navigator at nursing appointments with patients with diabetes at the health center, study the validity and reliability of Navigator, and characterize patients in each of the four CS groups. The hypothesis is that patients assigned to the network group will be older, more multimorbid and disabled than are patients in the self-acting group.

The detailed research questions are:

1. How user-friendly and time-consuming is Navigator as a web-based service at ordinary appointments, and does it add new issues to nurse-patient discussions (feasibility)? Does Navigator-based segmentation differ from current practice (intuition) in evaluating patient-specific health care service needs (criterion validity)?
2. Are questions studying the health status or everyday ability of patients to function relevant, sufficiently comprehensive, and comprehensible (content validity)?
3. Do all of Navigator’s items measure the same construct (internal consistency, reliability), and are all its questions necessary (construct validity)?
4. Is the CS-group segregation result repeatable after two-to-three weeks (test-retest) with the same professional (intrarater) or between professionals (interrater reliability)?
5. How are patients with diabetes segregated in a primary care setting, and what kinds of patients inhabit different CS groups?

## Methods

### Study Design

**Figure 2:**
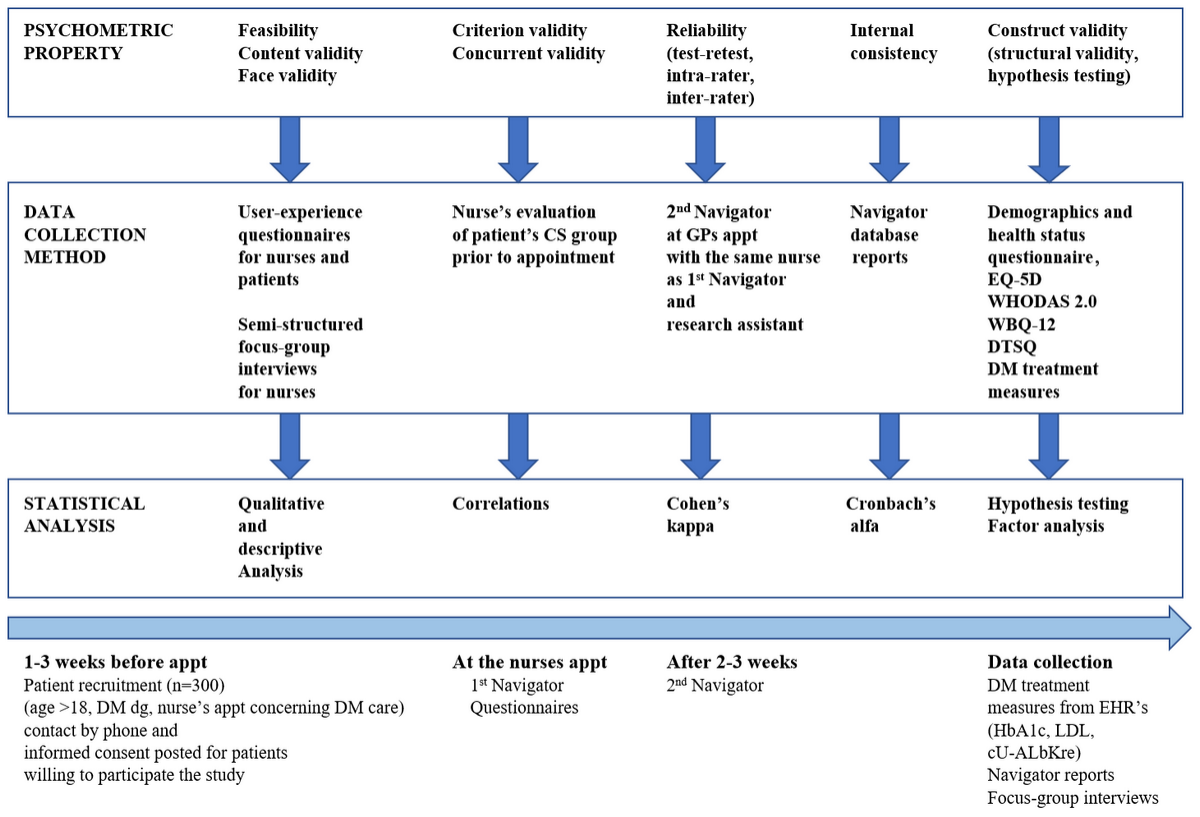
Psychometric properties of Navigator, data collection methods and statistical analysis used in study, and timeline of data collection process.

This mixed methods study combines qualitative and quantitative methods. Data collection is based on questionnaires for nurses and patients with diabetes, focus group interviews for nurses, the medical parameters of diabetes care, and Navigator reports from the Pirkanmaa Hospital District database (Figure 2). The study population of 300 patients and 12 nurses were recruited at Valkeakoski social and health center.

COSMIN criteria (consensus-based standards for selecting health-measurement instruments), and quality criteria for the measurement properties of health status questionnaires, were used as a methodological framework to study the content, construct, and criterion validity as well as the reliability of Navigator [43,44]. Patient-reported outcome measures have been developed to capture patients’ views on the effect of illnesses and symptoms in their everyday lives and assist communication and decision-making in care between doctors and patients [45]. In this study, Navigator is examined as a patient-reported outcome measure.

### Feasibility, Criterion and Concurrent Validity, Content and Face Validity

Data are gathered with questionnaires for patients and nurses and semistructured focus group interviews for nurses.

The self-generated user-experience questionnaire for patients studies the CS group and the patient’s opinion of the care pathway related to it through the Likert scale (from 1, meaning “complete agreement” to 5, meaning “complete disagreement”). Questions determine whether or not any help was needed, for example, from spouses, in answering Navigator’s questions. Seven statements study whether questions were easy or difficult or took too long to answer. Statements include that patients’ questions helped patients consider their life and everyday coping from a new perspective, that professionals’ questions helped patients understand their life situation, and wish to continue to use Navigator in planning their health care services. Furthermore, open questions study whether or not patients disagreed with their CS group allocation and whether new issues concerning their everyday life appeared in the discussion because of the Navigator’s questions.

Nurses fill out their user experience questionnaire after every patient with whom Navigator is used. It queries the time spent answering Navigator’s questions and whether or not patients required assistance to answer them. Both nurses’ and patients’ views of Navigator results are queried by way of the Likert scale. Open questions gather information on whether or not, and how, nurses’ or patients’ views differed regarding CS group allocation. The questionnaire also studies nurse intuition regarding CS group allocation with every patient. The questionnaire queries nurses’ previous contact with patients by phone or at appointments during the past year. These questions assess the criterion and the concurrent validity of Navigator.

Another questionnaire collects nurses’ experience and work history in primary care and their prior knowledge of customership strategies. Opinions are obtained using a Likert scale to address Navigator’s suitability for patient segregation and the usability of its results in coordinating care for patients with long-term conditions. Nine claims study whether or not the Navigator service was easy and suitable to use, and whether or not questions were too ambiguous or broad to be answered using the Visual Analog Scale (VAS). Users are also asked whether or not Navigator’s questions were difficult to understand, helped raise difficult issues with patients, and helped professionals understand the patients’ general care at a deep level. The final query is the plausibility of using Navigator and whether new issues appeared in the discussion via its use. Open questions specify if any of Navigator’s questions were difficult to understand or too broad. Additionally, nurses are asked to describe if using Navigator was difficult or time-consuming with certain kinds of patients. This questionnaire examines the feasibility and the content validity of Navigator.

Semistructured interviews for three focus groups of four-to-five nurses will be performed after two-to-three months of Navigator use for assessing a more detailed user experience to study the feasibility, the content, and the face validity of Navigator (Multimedia Appendix 1). Interviews will be recorded and transcribed verbatim by an official service provider, and the research team will analyze written material.

### Construct Validity

Construct validity consists of structural validity and hypothesis testing. Navigator measures two different constructs: patients’ coping in everyday life and patient health status. These two different constructs are evaluated separately. All answers to each Navigator question on the VAS of 1 to 10 are saved in a Pirkanmaa Hospital District database. This information is collected as “Navigator reports.”

Questionnaires examine data concerning patients’ demographics, medical condition, health-related quality of life, and coping in everyday life.

A self-generated questionnaire is used to collect patient demographics and health status. Patient gender, year of birth, marital status, education, and employment situation is queried. Health-related questions concern self-rated health, duration with a diabetes diagnosis, diabetes medication, knowledge of target values for individual diabetes care, other illnesses or chronic conditions, and medication. Also queried are smoking status, alcohol usage, height and weight, tools needed for physical disability assistance, and the receipt of disability benefits or care allowance for pensioners from the Social Insurance Institution of Finland.

The WHODAS 2.0 (World Health Organization Disability Assessment Schedule 2.0) self-administered 12-item version is used to study health-related disability during the last 30 days. Twelve questions concerning six domains of function (cognition, mobility, self-care, getting along, life activities, and participation) are answered on a 5-point scale ranging from “no difficulties” to “extreme difficulties or could not” [46].

EuroQol 5D (EQ-5D) is a generic health-status measure consisting of the EQ-5D-5L descriptive system and measuring five dimensions of health (mobility, self-care, usual activities, pain/discomfort, and anxiety/depression), with each dimension having five response levels from “no problems” to “extreme problems or unable to.” The EQ VAS measures the patient’s self-rated health on a vertical VAS whose endpoints are respectively “The best health you can imagine” and “The worst health you can imagine.” Patients are asked to evaluate their health on that day. The EQ-5D has Finnish Population Norms [47].

W-BQ12 is a general 12-item well-being questionnaire measuring negative well-being, energy, positive well-being, and general well-being. Patients evaluate their well-being during recent weeks on a 4-point scale from 0 (not at all) to 3 (all the time) [48].

Diabetes-treatment satisfaction questionnaires (DTSQs) are used to collect data on diabetes treatment satisfaction [49].

Medical parameters concerning diabetes care (HbA_1c_, low-density lipoprotein, albumin-creatinine, blood pressure) are evaluated annually according to the Finnish Current Care Guideline for diabetes [50]. Values are collected from patients’ medical records.

### Reliability

During the nurse’s appointment, Navigator is used for the first time, and the first CS group result is generated. Based on the patient’s condition and self-care capability, the nurse then evaluates the patient’s need for a physician appointment. Usually, a physician appointment is made with a physician-researcher within 3 weeks. At the beginning of the physician-researcher appointment, Navigator is used for the second time, with the same nurse, the physician, and a research assistant present (2018). All professionals complete their questionnaires independently, and three different results are generated.

### Study Population

Patients with diabetes (n=300 based on sample size calculation) were recruited in a primary care setting at Valkeakoski health center. Annual follow-up visits concerning diabetes care are carried out at appointments with first a nurse and then if needed, a physician within a month. Twelve nurses working at Valkeakoski health center at the time of data collection were recruited for the study.

### Inclusion and Exclusion Criteria

The inclusion criteria are age exceeding 18 years and a planned annual diabetes care follow-up appointment with a health center nurse. Exclusion criteria are patient disability preventing informed consent for participation in research (eg, Alzheimer disease, intellectual disability) and nonfluency with the Finnish language.

### Recruitment

Patients scheduled for an annual diabetes-control appointment with a nurse within three weeks were identified via the electronic patient record system. They were then contacted in advance by phone and informed about the study. Patients willing to read further about the study were sent the informed consent declaration form by post. Recruitment began in October 2018 and was completed in September 2019.

### Data Collection

Before the appointment with a recruited patient begins, the nurse is asked to evaluate the patient’s Navigator result intuitively based on knowledge of the patient and medical records (Figure 2). Intuition-based results will also be collected from patients refusing to participate in the study.

At the beginning of the appointment, the nurse confirms the patient’s participation, collects the signed consent form, and combines the patient’s social security number with their study identification code. All data is encoded for processing, protecting patients and nurses from identification. Patients also receive the study envelope containing study questionnaires. Then the Navigator is used for the first time.

Navigator is used for the second time at the physician-researcher appointment, and diabetes treatment measures are collected. Measures for patients who receive no physician-researcher appointments are collected separately from medical records.

Semistructured interviews for three focus groups of nurses will be performed after Navigator has been used for two or three months, after which Navigator reports are gathered from the database.

### Intervention

#### Development of the Navigator Service

The Navigator service was developed in collaboration with the Finnish Heart Association (FHA) and the Center of General Practice of Pirkanmaa Hospital District in the Kurkiaura project between 2011 and 2015. Navigator was produced by the FHA and is maintained by the Center of General Practice of Pirkanmaa Hospital District in Finland.

The development process began by describing different kinds of patients based on lifestyle studies. Patient stories describe how different individuals manage private matters and how capable they are of practicing self-care. Professional views of patient care, combined with patients’ personal views of self-care, helped to develop the idea of a fourfold table. Issues limiting self-care were studied in a survey of patients with acute myocardial infarct and led to the implementation of separate care pathways for different patient classes. The need then arose to develop a tool to segregate patients into different segments, considering both patient and professional perspectives.

Questions in quality-of-life measurements (eg, 15D) and the international classification of functioning, disability, and health (ICF), were familiarized and reflected upon with previous patient stories to develop themes for patients’ questions. Preliminary questions for patients were made and further developed in workshops with patients. A multiprofessional health care group suggested questions for professionals. Questions were agreed upon and further developed in meetings with multiprofessional health care experts (personal communication AR, LK).

#### Content of the Navigator Service

**Figure 3:**
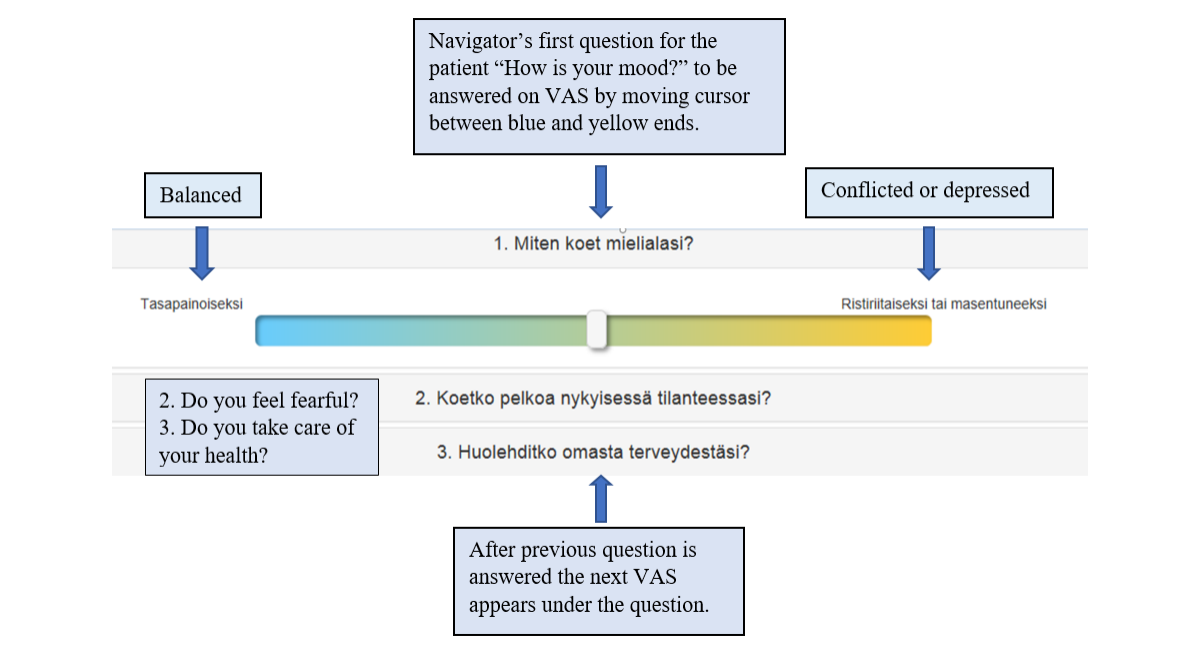
Navigator's first three questions for patients, showing VAS.

A 10-question patient and 8-question professional VAS are used (Multimedia Appendix 2, Figure 3). Answers on a scale of 1 to 10 are dichotomized at a certain cut point. The result of all answers is used to propose a CS group appropriate for each patient. Every CS group has its own care pathway, defining the focus of individual care plans, service and care coordinators, making appointments and contacting health care services, and alternatives to appointments and services typically included in certain pathways.

#### Customership Strategy Groups and Care Pathways

##### Self-Acting Group

Patients in the self-acting group competently manage everyday life with their illnesses and independently coordinate their health care. Health care services aim to support self-care in maintaining the ability to work and function. The individual health care plan for this group is focused on self-care. Planning alternative forms of health care services such as telemedicine options in contacts or as an alternative to appointments is essential with these patients who, according to pilots, make up the majority of all patients.

##### Community Group

The everyday life of patients in the community group is troubled, although their health status appears simple. Their individual health care plans focus on motivating and empowering their self-care, guiding them to peer group meetings, and building confidential health care relationships. The care coordinator could be a nurse who contacts the patient proactively, such as by phone, and organizes occasional appointments to ensure the appropriate use of health care services.

##### Cooperating Group

Patients in the cooperating group function in everyday life despite complicated health status. Their individual care plans focus on improving their ability to function and prevent complications and additional illnesses. The coordination of multiprofessional care and appointments is needed, and the coordinator is a health care professional. Electronic health care options could be used in contact with health care services.

##### Network Group

Patients in the network group are the most vulnerable and in need of intensive support. Their individual health care plans focus on maintaining the ability to function in everyday life at home and preventing hospitalization. Health care is proactive and usually multiprofessional. Services are coordinated by a professional, home visits are considered alternatives to appointments at the health center, and family support is essential.

### Sample Size

Sufficient patient population and sample size calculations are needed to compare outcomes of patient variables in different CS groups. The power calculation is based on the WHODAS 2.0 validation study of patients with chronic conditions in Europe. The results of a 36-item WHODAS 2.0 questionnaire gave a mean score of 24.8 (complex scoring scale 0.00-93.5) and a standard deviation of 19.3 [46]. No information on the 12-item WHODAS 2.0 questionnaire results in patients with diabetes was found for this purpose. Using a power of 80% and statistical significance of *P*=.05, a minimum of 30 patients are needed in each group, as the clinically significant difference between means of separate groups is assumed to be 14 on the WHODAS 2.0 scale (complex scoring 0.00-100). The effect size is moderate (d=0,725) [51]. In Navigator pilots, the smallest group (9%-10% of patients) was the network group, resulting in a total sample size of 300 patients. A sample size calculator was used for the calculation [52].

### Statistical Analysis

Feasibility, content, and face validity are examined with patient and professional user experience questionnaires and semistructured focus group interviews for professionals. Data analysis will be qualitative and descriptive. Qualitative thematic analysis will be used to analyze focus group interviews [53].

Criterion and concurrent validity are assessed, comparing nurse-intuited patient CS group allocation to the patient CS group allocation proposed by Navigator. Correlations can be calculated, and visible differences described.

Construct validity consists of structural validity and hypothesis testing. Navigator measures two constructs: the ability to cope in everyday life and patient health, and they are evaluated separately. Exploratory factor analysis is performed on each Navigator data report to evaluate how different factors are loaded. The assumption is that every question is important for determining either patient’s coping in everyday life or the patient’s health status. Descriptive statistics will be performed on patient values (demographic and health status, diabetes mellitus treatment measures, EQ-5D, WHODAS 2.0, WBQ-12, and DTSQ questionnaire responses) in different CS groups, and differences between groups will be studied by comparison of means. Linear-regression analysis is performed to assess which variables affect variation in CS-group allocation.

Intrarater, interrater, and test-retest reliability are analyzed by calculating Cohen kappa correlations between the first and second Navigator results obtained with the same nurse, in the presence of a physician and a research assistant. The internal consistency of items for both constructs will be analyzed by calculating Cronbach alpha.

### Ethical Approval

The Tampere University Hospital Ethics Committee approved this study’s ethical aspects in October 2018 (ETL R18070). Data collection at Valkeakoski Health Center was approved by head physician Myllynen in September 2018.

## Results

Descriptive results of strengths, difficulties, and time spent using the Navigator service during nurses’ appointments may help develop user instructions.

The results of patient segmentation into different CS groups may strengthen the results of previous pilot studies. The characteristics and differences of patients between groups are assumed to relate to Navigator’s results: patients managing well in everyday life (self-acting and cooperating groups) could have better results in WHODAS 2.0, EQ-5D, and WBQ-12 than do patients with difficulties in everyday life. Moreover, patients in self-acting and community groups, whose health status is simpler than that of cooperating- and network-group patients, could have fewer chronic conditions, require less medication, and enjoy more successful diabetes mellitus treatment measures.

Exploratory factor analysis may indicate identifiable factors in patients’ and professionals’ questions. This outcome of construct validity may be used in further developing Navigator’s items and be confirmed in future studies.

Correlations between the test and retest results of Navigator will be statistically analyzed using the Cohen kappa coefficient. The reliability outcome is moderate if values are 0.41-0.60, substantial if they are 0.61-0.80, and in almost perfect agreement if they are 0.81-1.00 [54]. In addition, how both patients’ and professionals’ questions measure the same construct helps assess the reliability and internal consistency of Navigator. Usually, internal consistency is high if Cronbach alpha is >0.7 (values between 0 and 1) [55].

## Discussion

### Principal Findings

This study is the first to assess the feasibility, validity, and reliability of Navigator, the Finnish innovation for patient segmentation. The study also examined the segregation of patients with diabetes into four customership groups in a primary care setting and the differences between patients in each group.

The Navigator service adds a patient's individual perspective of his/her ability to cope in everyday life to the methods of patient segmentation. EHR databases do not contain information pertinent to patients’ capacity to navigate between health services. Therefore, discussing these issues with patients is essential when individual and patient-centered care is planned.

Patient navigation is described as barrier reduction and as 'a bridge over gaps in services' method. Facilitating access to care, communicating with multiple agencies in fragmented health and social care, and navigator persons such as health care professionals, case managers, or laypersons have been reviewed in diverse settings [56,57]. The Navigator service standardizes individual recognition of patients with different needs, and separate care pathways developed for different CS groups help patients navigate between health services.

Note that this study does not assess the effectiveness or efficacy of the four separate care pathways that Navigator proposes. In the future, it will be essential to study how efficient Navigator is in patient navigation and how it affects patient health outcomes.

### Risks and Biases

Although several health centers in the Pirkanmaa region have been trialing the Navigator service with patients with different chronic conditions, it has yet to be properly implemented in health care use. Moreover, care pathways related to Navigator CS groups in different chronic conditions remain in development. Therefore, multicenter patient recruitment and regional data collection were unobtainable, and the study population was ultimately drawn from a single health center in Valkeakoski.

The study setting in health care services and particularly exclusion criteria may bias the study population and group segregation. Vulnerable patients may fail to familiarize themselves with the declaration of informed consent, leading to their refusal to participate in the study. In addition, vulnerable patients may not be treated in the health center’s ambulatory diabetic guidance clinic, and thus their proportion of the total participants may be low. Furthermore, making appointments at health centers requires an ability to personally deal with the health care system that many vulnerable patients may lack. Nurses’ intuitive evaluation of the customership of patients not participating in the study is also collected in order to assess this bias. Moreover, the self-selection bias of participants may impact results.

### Timetable and Publications

Data collection was completed in September 2019. Analysis of Navigator user experiences and focus group interviews were performed in Spring 2020. The feasibility assessment of Navigator is expected to be completed in 2020. Further analysis of the psychometric properties of Navigator is expected in 2021, and the results of patient segregation and outcomes in different CS groups are expected in 2022. Further publications are expected in 2020-2022, with a PhD thesis expected to be completed in 2022.

## Appendix

Multimedia Appendix 1 Content of the semi-structured focus-group interview for nurses.

Multimedia Appendix 2 Navigator service’s questions for patient and professional (translated in English by RR).

## Abbreviations

CS group: customership strategies group
DTSQ: Diabetes Treatment Satisfaction Questionnaire
EHR: electronic health record
EQ-5D: EuroQol 5D
FHA: Finnish Heart Association
ICF: International Classification of Functioning, Disability, and Health
WHODAS 2.0: World Health Organization Disability Assessment Schedule 2.0
VAS: Visual Analog Scale
