# Supplementary material for: Web-Based Patient Segmentation in Finnish Primary Care: Protocol for Clinical Validation of the Navigator Service in Patients With Diabetes
Source: JMIR Res Protoc. 2020 Nov 2;9(11):e20570. doi: 10.2196/20570 (PMC7669435; doi:10.2196/20570)
Supplement: Multimedia Appendix 1 [file resprot_v9i11e20570_app1.docx]

Table 1. Content of the semi-structured focus-group interview for nurses

| **General topics** | Navigator’s action  Navigator’s use at the appointment   - as a separate service or as part of conversation   Navigator’s appearance as digital service   - filling personal details - arrangement of questions above the VA scale - appearance of results page |
| --- | --- |
| **Patient observation** | Answering on VAS   - using computer interface themselves or indicating the answer otherwise   Appropriate text size of questions  Influence of VAS colours on answers  Answers spreading on VAS  Lack of numbers affecting answering on scale |
| **Nurses’topics** | Answers spreading on VAS  Each patient’s and professional’s question is discussed separately:   - thoughts, problems or uncertainties arising from questions or answering options - comprehensibility of answering options at the ends of VA scale - important issues related to coping in daily life missing from questionnaire |
