# Supplementary material for: Web-Based Patient Segmentation in Finnish Primary Care: Protocol for Clinical Validation of the Navigator Service in Patients With Diabetes
Source: JMIR Res Protoc. 2020 Nov 2;9(11):e20570. doi: 10.2196/20570 (PMC7669435; doi:10.2196/20570)
Supplement: Multimedia Appendix 2 [file resprot_v9i11e20570_app2.docx]

Table 2. Navigator service’s questions for patient and professional (translated in English by RR)

| **Navigator’s dimension of patient’s ability to function in every-day life** | |  | |
| --- | --- | --- | --- |
| **Patient’s question** | **Endings of VAS** | | |
|  |  | | |
| How is your mood? | Balanced ̶ conflicted or depressed | | |
| Do you feel fearful? | feel calm ̶ feel fearful | | |
| Do you take care of your health? | health issues always matter ̶ other things matter | | |
| Are you able to manage your illness? | yes ̶ no | | |
| How do you manage in everyday life? | I manage well ̶ I do not manage | | |
| How is your ability to move? | I am able to move ̶ I am unable to move | | |
| Have you friends or relatives to support you? | Friends or relatives support me if needed ̶  I require assistance | | |
| Do some of the following sentences describe your situation?  (tick the box if YES) | Others depend on me for care  I am concerned about my coping in everyday life at home  I have financial worries  I have other worries | | |
| Do you know how to seek professional help if needed? | I manage with the health service ̶ I am unfamiliar with the health-service system | | |
| Do you know how to use electronic services? | yes ̶ no | | |
|  |  | | |
| **Navigator’s dimension of patient’s health status or the degree of disease and treatment** | | |  |
| **Professional’s question** | **Endings of VAS** | | |
|  |  | | |
| Patient’s health status | simple ̶ complicated | | |
| Professionals needed in patient’s care | few ̶ many | | |
| Is active self-care of illnesses needed? | a little ̶ a lot | | |
| Medication regimen | straightforward ̶ complicated | | |
| Are social or mental factors, drug or alcohol abuse, dementia or sense disorders affecting care? | a little ̶ a lot | | |
| Patient has diagnoses/risks/problems | few ̶ many | | |
| Care balance | good ̶ poor | | |
| Carrying out health care | simple ̶ complicated | | |
